# PLGA Containing Human Adipose-Derived Stem Cell-Derived Extracellular Vesicles Accelerates the Repair of Alveolar Bone Defects via Transfer of CGRP

**DOI:** 10.1155/2022/4815284

**Published:** 2022-06-11

**Authors:** Yang Yang, Bo Zhang, Yufan Yang, Bibo Peng, Rui Ye

**Affiliations:** ^1^State Key Laboratory of Oral Diseases and National Clinical Research Center for Oral Diseases, West China Hospital of Stomatology, Sichuan University, Chengdu 610041, China; ^2^Department of Orthodontics, West China Hospital of Stomatology, Sichuan University, Chengdu 610041, China

## Abstract

Calcitonin gene-related peptide (CGRP) is an important neuropeptide expressed in the nerve fibers during bone repair. Here, we aimed to pinpoint the role of CGRP in the osteogenic differentiation property of human periodontal ligament stem cells (hPDLSCs) and the resultant repair of alveolar bone defect. The key factor related to the osteogenic differentiation of hPDLSCs was retrieved from the GEO database. After extraction from hADSCs (hADSC-EVs) and identification, EVs were subjected to coculture with hPDLSCs, in which the expression patterns of CGRP and osteogenic differentiation marker proteins (ALP, RUNX2, and OCN), as well as ALP activity, were detected. A novel cell-free tissue-engineered bone (TEB) comprised of PLGA/pDA and hADSC-EVs was implanted into the rats with alveolar bone defects to evaluate the repair of alveolar bone defects. CGRP was enriched in hADSC-EVs. hADSCs delivered CGRP to hPDLSCs through EVs, thereby promoting the osteogenic differentiation potential of hPDLSCs. The PLGA/pDA-EV scaffold released EVs slowly, and its implantation into the rat alveolar bone defect area significantly induced bone defect repair, which was reversed by further knockdown of CGRP. In conclusion, our newly discovered cell-free system consisted of hADSC-EVs, and PLGA/pDA scaffold shows promising function in repairing alveolar bone defects.

## 1. Introduction

Bone defects show close linkage with a significant pressure of disease, with the treatment outcomes astricted by high occurrence of complication and reoperation, along with dismal functional outcomes [1]. Human periodontal ligament stem cells (hPDLSCs) show potential to treat regeneration of dental tissues due to their potential angiogenetic, immunoregulatory and anti-inflammatory properties [2]. The osteogenic differentiation of hPDLSCs is highly correlated with alveolar bone repair and periodontal regeneration [3, 4]. Elucidating the mechanism of osteogenic differentiation potentials of hPDLSCs thus facilitates the development of alveolar bone repair approaches.

Adipose-derived stem cells (ADSCs) are attractive candidates to repair damaged organs and tissues as they can maintain self-renewal and induce multidifferentiation potential by releasing paracrine factors and extracellular vesicles (EVs) [5]. EVs include exosomes and microvesicles and exert crucial function in diseases and malignancies [6, 7]. Calcitonin gene-related peptide (CGRP), which is a neuropeptide to regulate the performance of bone tissues, induces osteogenic differentiation capability of BMSCs in the context of osteoporotic fracture [8]. The CGRP overexpression in ADSCs enhances the potential of ADSCs to differentiate into osteoblasts in vitro and the resultant repair of rat radial bone defects in vivo [9]. Meanwhile, inhibition of CGRP blunts the enhanced critical size bone defect repair induced by magnesium (Mg) nail during the distraction osteogenesis process [10]. Poly (lactic-co-glycolic acid) (PLGA) nanoparticles serve as the promising delivery carriers for the CGRP and can facilitate the therapeutic effects of CGRP [11]. PLGA is one of the extensively researched synthetic biodegradable polymers, and PLGA micro/nanoparticles prevent instant degradation in macromolecules while maintaining tunable release rate and profile, ascribed to their potent biocompatibility and controllable biodegradability [12, 13]. A nanocomposite scaffold of grafted hydroxyapatite (g-HA)/PLGA bears the responsibility to trigger mineralization and bone formation, facilitating the repair of the critical radius defects in rabbits [14]. In addition, the polydopamine-assisted BFP-1-immobilized PLGA (pDA-BFP-1-PLGA) scaffold can promote the bone formation in nude mice [15]. We therefore proposed a hypothesis that CGRP delivered by hADSC-derived EVs (hADSC-EVs) loaded with PLGA could serve to facilitate repair of alveolar bone defects.

## 2. Materials and Methods

### 2.1. Ethics Statement

Experimental procedures regarding human were implemented with the approval of the Clinical Ethics Committee of West China Hospital of Stomatology, Sichuan University and in the light of the *Declaration of Helsinki*. All participants signed informed consent documentation before sample collection. Animal experiments were ratified by the Animal Ethics Committee of West China Hospital of Stomatology, Sichuan University, and performed according to the Guide for the Care and Use of Laboratory animals published by the US National Institutes of Health (approval number: WCHS-IRB-S-006-A01-V002.02).

### 2.2. Bioinformatic Analysis

hPDLSC osteogenic differentiation-related gene expression dataset GSE53929 (contained 9 control samples and 9 CEMP1-induced osteogenic differentiation samples) was attained from the Gene Expression Omnibus (GEO) database with the platform of GPL6244 (HuGene-1_0-st) Affymetrix Human Gene 1.0 ST array (transcript (gene) version). Differential analysis was implemented utilizing GEO2R tool with *p* < 0.05 set as the threshold. Osteogenic differentiation-related genes were searched from the GeneCards database, and the top 100 genes with correlation scores were selected for further analysis. The jvenn online tool was applied to analyze the overlap between the differentially highly expressed genes and osteogenic differentiation-related genes. The interaction of the obtained candidate genes was checked with the GeneMANIA tool.

### 2.3. Cell Culture and Treatment

hADSCs (CP-H202, Wuhan Procell Life, Wuhan, China) were cultured in proliferation medium (PM) appended to Dulbecco's Modified Eagle's Medium (11054020, Gibco, Grand Island, NY), 10% fetal bovine serum (FBS; F8687, Sigma-Aldrich, St Louis, MO), 100 U/mL penicillin, and 100 *μ*g/mL streptomycin (HyClone Laboratories, Logan, Utah) in a 5% CO_2_ incubator with 100% relative humidity at 37°C.

A lentivirus package kit was used to package lentiviral particles carrying short hair RNAs (Shanghai GenePharma Co., Ltd., Shanghai, China) against CGRP (shCGRP) and negative control (shNC) into hADSCs (SCSP-502, Cell Bank of the Type Culture Collection, Chinese Academy of Sciences, Shanghai, China). After 48 h, the virus supernatant was gathered and concentrated. Upon reaching approximately 50% confluence, the cells were transduced with lentivirus and screened with 10 *μ*g/mL puromycin 48 h after transduction, maintaining for at least 1 week to select stably transduced cell lines. Three CGRP shRNA sequences (shCGRP-1: 5′-GCACATACACGCAGGACTTCA-3′; shCGRP-2: 5′-GCAGGACTTCAACAAGTTTCA-3′; shCGRP-3: 5′-GCGGTAATCTGAGTACTTGCA-3′) were designed, and the most effective sequence was pinpointed for further experiments.

### 2.4. Isolation and Culture of hPDLSCs

Healthy teeth were taken from 10 healthy participants who underwent tooth extractions at West China Hospital of Stomatology, Sichuan University, due to orthodontics. The middle third of the roots was selected, and the periodontal ligament tissues were scraped with a blade. The tissue fragments were submitted to digestion utilizing 3 mg/mL type I collagenase (SCR103, Sigma-Aldrich) in a 37°C water bath for 30 min. Next, cell culture was implemented in *α*-minimum essential medium (*α*-MEM) appended to 10% FBS and 1% streptomycin and penicillin in a 37°C incubator with 5% CO_2_, with the medium refreshed every 3 days. Cells at passage 3–4 were chose for all experiments. To induce bone formation, hPDLSCs were placed in a 6-well petri dish (2 × 10^4^ cells/well). When reaching 80% confluence, cell culture was implemented in osteogenic medium (OM) with 5 mM *β*-glycerophosphate (G9422, Sigma-Aldrich), 50 *μ*g/mL ascorbic acid (BP461, Sigma-Aldrich), and 10 nM dexamethasone.

### 2.5. Identification of hPDLSCs

Immunofluorescence staining: hPDLSCs were inoculated in a 24-well plate (1 × 10^5^ cells/well). After 2 days, the cells were fixed with precooled 4% paraformaldehyde (P1110, Solarbio, Shanghai, China) for 20 min and reacted with 0.1% Triton X-100 (T109026, Shanghai Aladdin Bio-Chem Technology Co., Ltd., Shanghai, China) for 15 min. Subsequently, the cells were treated with 1% bovine serum albumin (BSA) for 30 min to block nonspecific binding and probed with primary antibodies against cytokeratin (ab52625, 1 : 200, Abcam Inc., Cambridge, UK) and vimentin (ab92547, 1 : 250, Abcam) for 1 h and then with secondary antibody Alexa Fluor 488 (green)-conjugated goat antibody (ab150113, 1 : 100, Abcam) for 1 h. Following three washes with PBS, the cells were stained with Hoechst 33342 (B2261, 1 : 10000, Sigma-Aldrich) and observed under a fluorescence microscope.

Detection of multiple differentiation ability: hPDLSCs were cultured in 6-well plates (1 × 10^6^ cells/well) without inducers. Upon reaching 80% confluence, the cells were cultured with osteogenic differentiation induction medium (StemPro Osteogenesis Differentiation Kit, A1007201, Thermo Fisher Scientific Inc., Waltham, MA) for 2 weeks for osteogenic differentiation and then identified by Alizarin Red S (ARS) staining and alkaline phosphatase (ALP) staining. For the adipogenic differentiation, the cells were cultured with adipogenic medium (StemPro Adipogenic Differentiation Kit, A1007001, Thermo Fisher Scientific) for 14 days and then stained with oil red O. For chondrogenic differentiation, cells were cultured in a 24-well cell culture dish at a density of 1 × 10^5^ with chondrogenic medium (StemPro Chondrogenic Differentiation Kit, A1007101, Thermo Fisher Scientific). After 2 weeks of induction, the cells were fixed with 4% paraformaldehyde, embedded in paraffin, and cut into 5 *μ*m-thick sections, followed by identification with Alcian blue staining (Cyagen, HUXMA-90041).

### 2.6. Isolation and Identification of EVs

EVs were extracted from hADSC conditioned medium in vitro. Before use, the FBS in the culture medium was ultracentrifuged at 100000 g for 12 h to remove bovine EVs. Cells were seeded in 6-well plates (2 × 10^5^ cells/well). After adherence to the wall overnight, the cells were cultured in the renewed EV-free serum for 48 h. Cell supernatant was collected, and EVs were extracted by differential centrifugation. All centrifugation procedures were conducted at 4°C, and the remaining procedures were operated on ice. Specifically, cells were centrifuged at 500 g for 15 min, at 2000 g for 15 min, and 10000 g for 20 min to remove dead cells and debris. After filtration utilizing a 0.22 *μ*M filter, the cells were centrifuged at 110000 g for 70 min and then resuspended in PBS at 4°C. Next, the cells were untracentrifuged at the same conditions. The pellet was stored at -80°C for later use or used immediately.

Transmission electron microscopy (TEM) was chose for observation of morphology of EVs. The expression of EV specific surface markers (Hsp70 [ab5442, 1 : 1000, Abcam], CD63 [ab134045, 1 : 1000, Abcam], Histone H3 [ab1791, 1 : 1000, Abcam], and *α*-tubulin [ab52866, 1 : 1000, Abcam], with Ponceau red used as an internal reference) was tested with the help of western blot analysis to ascertain the characteristics of EVs. In addition, nanoparticle tracking analysis (NTA) with a NanoSight LM10 instrument (NanoSight, Ltd., Minton Park, UK) was used to pinpoint the size distribution of EVs.

### 2.7. Confocal Microscope

The EVs were labeled with PKH67 red fluorescent kit (Sigma-Aldrich) [16]. The nuclei were stained with Hoechst (Molecular Probes), and the results were analyzed with a confocal microscope.

### 2.8. ALP Activity Detection

hPDLSCs were plated in 96-well plates (1 × 10^5^ cells/well). After 14 days of induction, ALP detection kit (NanJing JianCheng Bioengineering Institute, Nanjing, China) was employed for testing ALP activity. With p-nitrophenyl phosphate regarded as the substrate, the ALP activity was tested at 405 nm. The ALP development kit (Beyotime) was chose to develop the ALP in cells.

### 2.9. ARS Staining

hPDLSCs were plated in a 24-well plate (2 × 10^5^ cells/well). At 14 days after induction, the cells were stained with 0.2% ARS (Solarbio, pH = 8.3) according to the kit (Sigma-Aldrich) at 24-26°C for 10 min, washed with PBS, and put under an inverted microscope to observe the level of matrix mineralization. For detection of calcium deposition, the ARS dye in hPDLSCs was collected utilizing 400 *μ*L of 10% (w/v) cetylpyridinium sodium chloride solution in 10 mM sodium phosphate solution for 10 min and then figured at 562 nm on an ultraviolet-visible (UV-Vis) spectrometer.

### 2.10. Determination of the Optimal EV Concentration

EVs were isolated from the hADSC supernatant 14 days after OM induction. At the same time, Pierce protein determination kit (Thermo Fisher Scientific) was chose to determine the concentration of EVs. hPDLSCs were treated with EVs of different concentrations to determine the osteoinductive effect of EVs on hPDLSCs.

### 2.11. Construction of a New Type of Cell-Free Tissue-Engineered Bone (TEB)

TEB was constructed using EVs and PLGA/pDA scaffolds as previously constructed methods [17] utilizing cylindrical PLGA (4 mm in diameter and 2 mm in height; lactide/glycol ester: 50/50, Shandong Academy of Medical Sciences, Shandong, China). Pierce protein assay kit (Thermo Fisher Scientific) was employed to quantify the release of EVs. The surface morphology of the material was observed utilizing a Hitachi S-4800 field emission scanning electron microscope (Hitachi, Tokyo, Japan).

### 2.12. RNA Isolation and Quantitation

Total RNA from cells was purified with RNeasy Plus Mini Kit (Qiagen, Gathersburg, MD, USA). The total RNA was reverse transcribed into complementary DNA (cDNA) with a PrimeScript RT reagent Kit (Promega, Madison, WI, USA). Reverse transcription quantitative polymerase chain reaction (RT-qPCR) was implemented utilizing SYBR Green Master Mix (Life Technologies). GAPDH served as the loading control, and the fold changes were calculated utilizing relative quantification (the 2^-*ΔΔ*Ct^ method). The primer sequences are shown in Supplementary Table 1.

### 2.13. Western Blot Analysis

Total protein was extracted from cells and tissues with SDS lysis buffer (Beyotime), with the concentration measured utilizing a bicinchoninic acid kit (20201ES76, YEASEN Biotechnology Co., Ltd., Shanghai, China). After separation using 8% sodium dodecyl sulfate polyacrylamide gel electrophoresis, the sample was submitted to electrotransfer onto polyvinylidene fluoride membranes (Millipore, Billerica, MA, USA) which were blocked using 5% blocking solution with skimmed milk powder and underwent overnight incubation at 4°C with primary rabbit antibodies against CGRP (ab189786, 1 : 1000, Abcam), RUNX2 (ab76956, 1 : 1000, Abcam), OCN (ab93876, 1 : 500, Abcam), ALP (ab229126, 1 : 1000, Abcam), CD45 (ab40763, 1 : 5000, Abcam), CD73 (ab133582, 1 : 5000, Abcam), and CD90 (ab92574, 1 : 1000, Abcam) as well as 1 h-incubation with horseradish peroxidase-labeled secondary antibody goat anti-rabbit IgG (ab150077, 1: 1000, Abcam) at ambient temperature. The immunocomplexes on the membrane were visualized utilizing enhanced chemiluminescence (ECL) reagent (ECL808-25, Biomiga, USA) at ambient temperature for 1 min, and band intensities were quantified with the help of Image Pro Plus 6.0 software (Media Cybernetics, Silver Springs, MD, USA).

### 2.14. Construction of a Rat Model of Alveolar Bone Defects

Forty six-week-old male Wistar rats (Beijing Vital River Laboratory Animal Technology Co., Ltd., Beijing, China) were housed in the specific pathogen-free environment at 22-25°C and 60-65% humidity under a 12-h light/dark cycle (drink and eat freely). The rats were acclimated for one week before experiment. The rats were under general anesthesia, and the alveolar bone was exposed through a medical incision. Next, the rats underwent surgery to repair bilateral maxillary alveolar bone defects (3 mm in length, 1.5 mm in width, and 1.5 mm in depth). The scaffold in different groups was implanted into the defect, and the rats were divided into 4 groups (10 rats in each group): control, PLGA/pDA (PLGA scaffold coated with pDA), PLGA/pDA-EVs (PLGA/pDA scaffold plus EVs), and PLGA/pDA-EVs-shCGRP (PLGA/pDA scaffold plus EVs-shCGRP). After the operation, all rats were kept in a warm place until awakened and allowed to move freely in the cage. After 6 weeks, rats were euthanized by carbon dioxide inhalation, and the entire alveolar was taken. All specimens were washed 3 times with PBS and fixed with 4% paraformaldehyde at 4°C for 48 h.

### 2.15. Micro-CT Scanning

A micro-CT scanner (SCANCO *μ*CT50, Muttenz, Switzerland) was used to test the newly formed bone tissues in the rat alveolar defect. Mimics 17.0 software (Materialise, Leuven, Belgium) was chose to collect three-dimensional images and compute the bone density of the new bone-like tissue.

### 2.16. Histopathological Evaluation

The tissue was decalcified with 10% EDTA (pH = 7.4) for 1 month. The samples were then subjected to hematoxylin and eosin (HE) staining and Masson's trichrome staining [18]. The sections were scanned utilizing a tissue scanning equipment (Aperio, ScanScope XT, USA).

### 2.17. Immunofluorescence Staining

The undecalcified semithin sections were embedded with London resin, and all samples were immunofluorescently labeled with white resin. The sample was blocked with 3% BSA-PBST for 1 h and immunostained with primary human CGRP monoclonal antibody (ab81887, 1 : 100, Abcam) and then with Alexa Fluor 488 green fluorescent conjugated goat antibody (ab150113, 1 : 100, Abcam). The sections were analyzed by CLSM (LSM800) using a bright field optical microscope connected to a high-resolution digital camera (DFC425B).

### 2.18. Statistical Analysis

All data were analyzed using SPSS 18.0 statistical software (SPSS Inc., New York, IL, USA). The measurement data were described as mean ± standard deviation. Unpaired *t*-test (two group comparison) and one-way analysis of variance (ANOVA) and Tukey's multiple comparisons test (multiple group comparison) were chose in our study. A value of *p* < 0.05 was concluded as statistically significant. Each experiment was implemented three times independently.

## 3. Results and Discussions

### 3.1. CGRP Is Enriched in the hADSC-EVs

**Figure 1:**
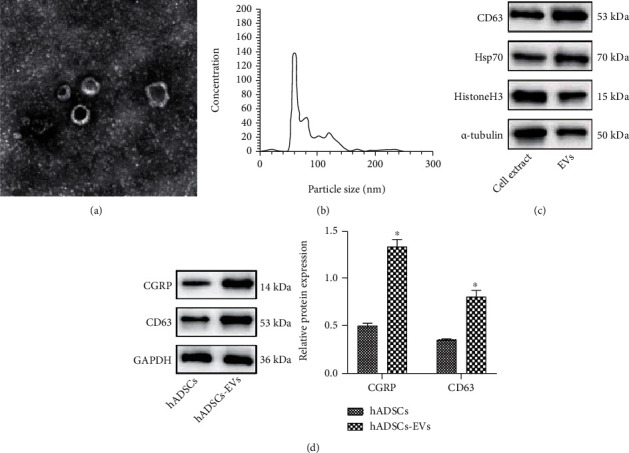
Identification of hADSC-EVs and CGRP expression in hADSC-EVs. (a) Microscopic views of the morphology of EVs under a TEM (scale bar = 100 nm). (b) EV size distribution measured by NTA. (c) Western blot analysis of EV marker proteins (CD63, Hsp70, and histone H3) and nonmarker *α*-tubulin. (d) Western blot analysis of CGRP protein in hADSCs and hADSC-EVs, *N* = 3.

We first isolated EVs from hADSCs, and TEM analysis results showed that EVs exhibit the characteristic cup-shaped morphology (Figure 1(a)). The results of NTA analysis showed that the size distribution of EVs ranged between 35 and 172 nm (Figure 1(b)). Moreover, Western blot analysis results revealed the presence of EV specific markers CD63 and Hsp70 in hADSC-EVs, but tubulin (cytoplasmic marker) or histone H3 (nuclear marker) was not present (Figure 1(c)). The above results indicate the successful isolation of hADSC-EVs.

The exoRBase online database (http://www.exorbase.org/exoRBase) predicted that CGRP (also known as CALCA) mainly existed in blood circulating EVs. In addition, CGRP was found to be significantly enriched in hADSC-EVs (Figure 1(d)). The above results indicate that CGRP is enriched in the hADSC-EVs.

### 3.2. hPDLSCs Uptake hADSC-EVs to Increase the CGRP Expression

**Figure 2:**
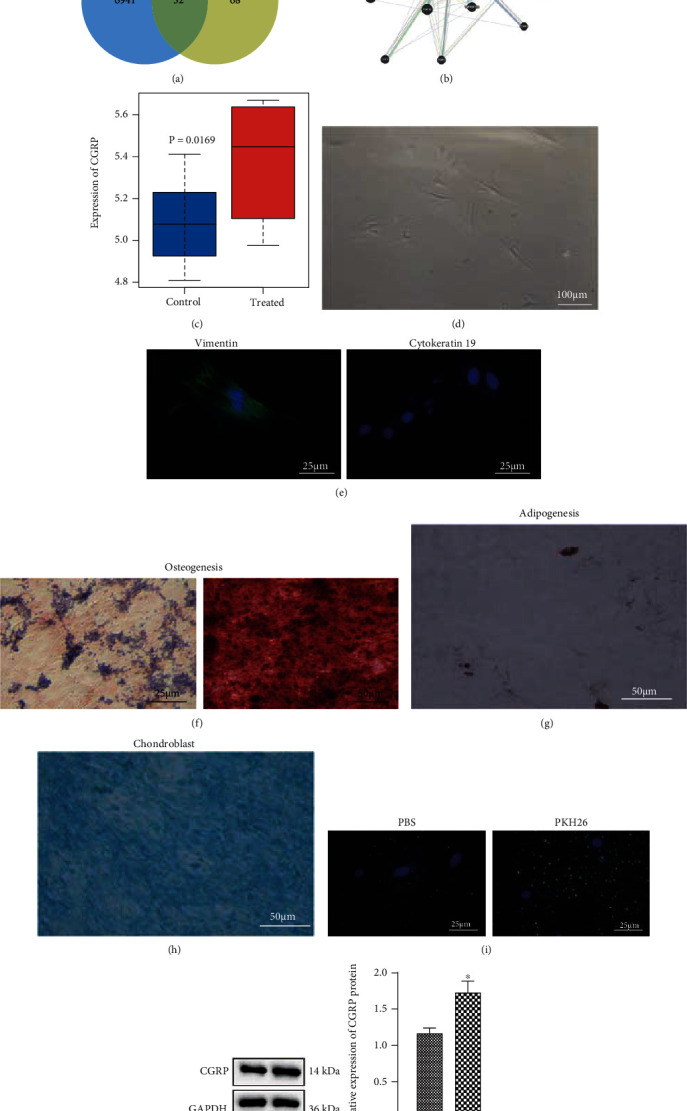
hPDLSCs uptake hADSC-EVs to increase the CGRP expression. (a) Venn diagram of the significantly highly expressed genes in the GSE53929 dataset and the top 100 genes related to hPDLSC osteogenic differentiation obtained from the GeneCards database. (b) Interaction network of candidate genes. (c) CGRP expression in the GSE53929 dataset. (d) The morphology of single colony forming unit of hPDLSCs (scale bar = 100 *μ*m). (e) Immunofluorescence staining of vimentin and cytokeratin proteins in hPDLSCs (scale bar = 50 *μ*m). (f) Osteogenic differentiation of hPDLSCs determined by ALP staining and ARS staining (scale bar = 50 *μ*m). (g) Adipogenic differentiation of hPDLSCs determined by Oil Red O staining (scale bar = 50 *μ*m). (h) Chondrogenic differentiation ability of hPDLSCs determined by Alcian blue staining (scale bar = 50 *μ*m). (i) Fluorescence microscopic images of hADSC-EVs (red dots) labeled by PKH26 internalized by hPDLSCs. hPDLSCs were stained with phalloidin Alexa Fluor (green), and Hoescht (blue) was used for nuclear counterstaining (scale bar = 25 *μ*m). (j) Western blot analysis of CGRP protein in hPDLSCs cocultured with hADSC-EVs. ^∗^*p* < 0.05, compared with the control samples or control hPDLSCs, *N* = 3.

Differential analysis on the GSE53929 dataset suggested 6953 significantly highly expressed genes (Supplementary Figure 1). Meanwhile, the top 100 genes related to hPDLSC osteogenic differentiation were obtained from the GeneCards database. Following Venn diagram analysis of these genes, 32 candidate genes were found at the intersection (Figure 2(a), Supplementary Table 2).

Analysis on the interaction network of candidate genes by the GeneMANIA tool indicated that six genes had higher interaction scores, including DMP1 (score = 0.867194116), SOST (score = 0.814230232), SP7 (score = 0.786778992), IBSP (score = 0.758063414), MEN1 (score = 0.737518121), and CALCA (score = 0.733867971) (Figure 2(b)). Existing evidence has confirmed the correlation between CGRP and osteogenic differentiation of hPDLSCs [19, 20]. CGRP was also predicted to be upregulated in the GSE53929 dataset (Figure 2(c)). Therefore, CGRP was selected as the target gene for follow-up research.

Under an optical microscope, hPDLSCs showed a typical fusiform morphology (Figure 2(d)). The results of immunofluorescence staining showed that hPDLSCs positively expressed the MSC marker vimentin and negatively expressed the epithelial marker cytokeratin (Figure 2(e)). Western blot analysis results showed that CD73 and CD90 were positively expressed in the hPDLSCs while the negative marker CD45 was not expressed (Supplementary Figure 2). In addition, the results of ALP and ARS staining confirmed the osteogenic differentiation ability of hPDLSCs (Figure 2(f)). Moreover, the lipid droplets in the cytoplasm were stained red, confirming the adipogenic differentiation ability of hPDLSCs (Figure 2(g)). Blue collagen staining was also seen, confirming the chondrogenic differentiation ability of hPDLSCs (Figure 2(h)). These results demonstrate the successful isolation of hPDLSCs.

PKH26-labeled EVs were coincubated with hPDLSCs. Fluorescence microscopic images showed that hADSC-EVs (red dots) labeled by PKH26 were gradually internalized by hPDLSCs (Figure 2(i)). As shown in Figure 2(j), elevated CGRP was seen in hPDLSCs cocultured with hADSC-EVs. The aforementioned results suggest that hPDLSCs can internalize hADSC-EVs, thereby increasing the expression of CGRP in hPDLSCs.

### 3.3. hADSC-EVs Promote Osteogenic Differentiation Potentials of hPDLSCs In Vitro

**Figure 3:**
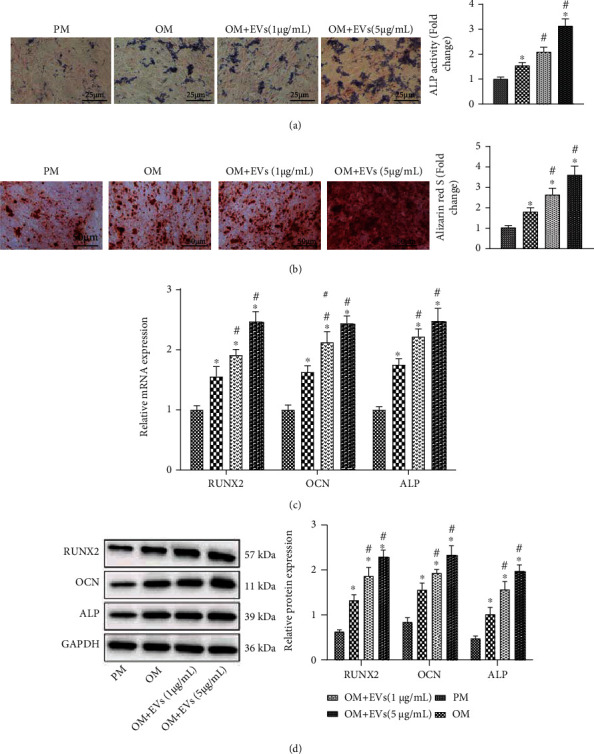
Promoting effect of hADSC-EVs on the osteogenic differentiation of hPDLSCs in vitro. hPDLSCs cultured in OM were coincubated with EVs (1 *μ*g/mL or 5 *μ*g/mL). (a) ALP staining analysis of ALP activity in hPDLSCs (scale bar = 25 *μ*m). (b) ARS staining of cellular matrix mineralization in hPDLSCs (scale bar = 50 *μ*m). (c) Expression of RUNX2, ALP, and OCN determined by RT-qPCR in hPDLSCs. (d) Western blot analysis of RUNX2, ALP, and OCN proteins in hPDLSCs. ^∗^*p* < 0.05, compared with hPDLSCs cultured in PM. #*p* < 0.05, compared with hPDLSCs cultured in OM, *N* = 3.

We then aimed to determine whether hADSC-EVs affect the osteogenic differentiation property of hPDLSCs. At 14 days after induction of hADSC-EVs, hPDLSCs cultured in OM showed enhanced ALP activity (Figure 3(a)). In addition, the cellular matrix mineralization was found to be increased in the presence of EV induction (Figure 3(b)). Similarly, compared with OM, the mRNA and protein expression of osteogenesis-related genes (RUNX2, ALP and OCN) was much higher in hPDLSCs treated with OM + EVs, in an EV dose-dependent manner (Figures 3(c) and 3(d)).

### 3.4. hADSC-EVs Promote the Osteogenic Differentiation Capability of hPDLSCs In Vitro by Delivering CGRP

**Figure 4:**
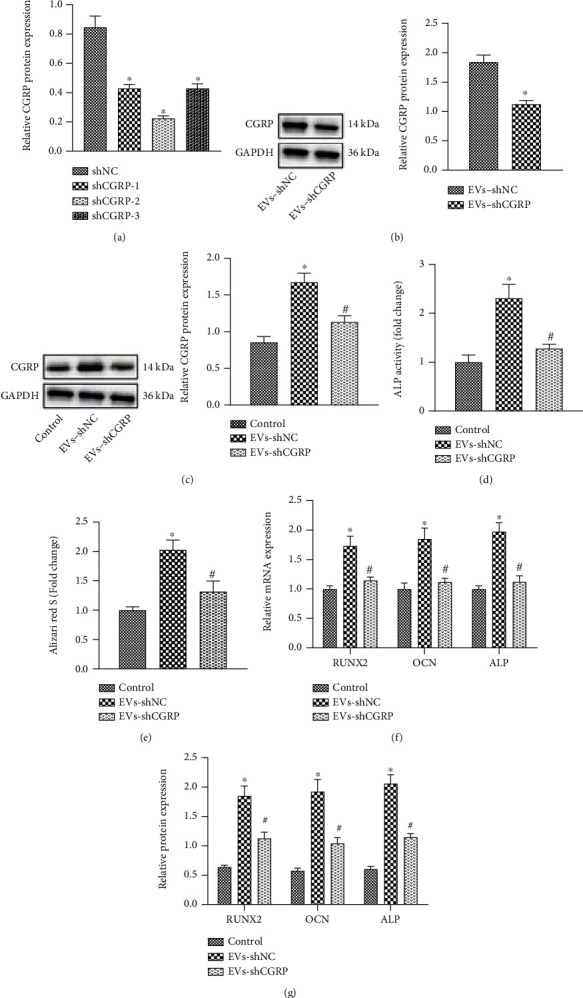
hADSC-EVs induce the osteogenic differentiation of hPDLSCs by delivering CGRP. (a) Western blot analysis of CGRP protein in hADSCs treated with shCGRP-1, shCGRP-2, and shCGRP-3. (b) Western blot analysis of CGRP protein in hADSC-EVs EVs-shCGRP. (c) Western blot analysis of CGRP protein in the presence of EVs-shNC or EVs-shCGRP. hPDLSCs were coincubated with EVs-shNC or EVs-shCGRP. (d) ALP staining analysis of ALP activity in hPDLSCs. (e) ARS staining of cellular matrix mineralization in hPDLSCs. (f) Expression of RUNX2, ALP, and OCN determined by RT-qPCR in hPDLSCs. (g) Western blot analysis of RUNX2, ALP, and OCN proteins in hPDLSCs. ^∗^*p* < 0.05, compared with shNC- or EV-shNC-treated hADSCs or control hPDLSCs. #*p* < 0.05, compared with EV-shNC-treated hPDLSCs, *N* = 3.

The mechanism of hADSC-EVs that promote the osteogenic differentiation capability of hPDLSCs was our next focus. The expression of CGRP in hADSCs was knocked down using lentivirus carrying shCGRP-1, shCGRP-2, and shCGRP-3, of which the shCGRP-2 showing the superior knockdown efficiency (Figure 4(a)) was selected for subsequent isolation of EVs. A decline in the CGRP expression was seen in EVs from shCGRP-treated hADSCs (EVs-shCGRP) (Figure 4(b)). Coculture data indicated the increase of CGRP expression in hPDLSCs cultured in OM for 14 days following EVs-shNC treatment while it was reduced after EVs-shCGRP treatment (Figure 4(c)). In addition, EVs-shNC treatment led to an increase in the ALP activity, which was reversed in the presence of EVs-shCGRP (Figure 4(d)). Consistently, cell matrix mineralization was augmented in the presence of EVs-shNC while it was attenuated following EVs-shCGRP treatment (Figure 4(e)). Furthermore, enhancement in the expression of RUNX2, ALP, and OCN was observed upon treatment with EVs-shNC while knockdown of CGRP caused opposite results (Figures 4(f) and 4(g)).

### 3.5. In Vitro Morphological Analysis of TEB and Observation of EV Release

**Figure 5:**
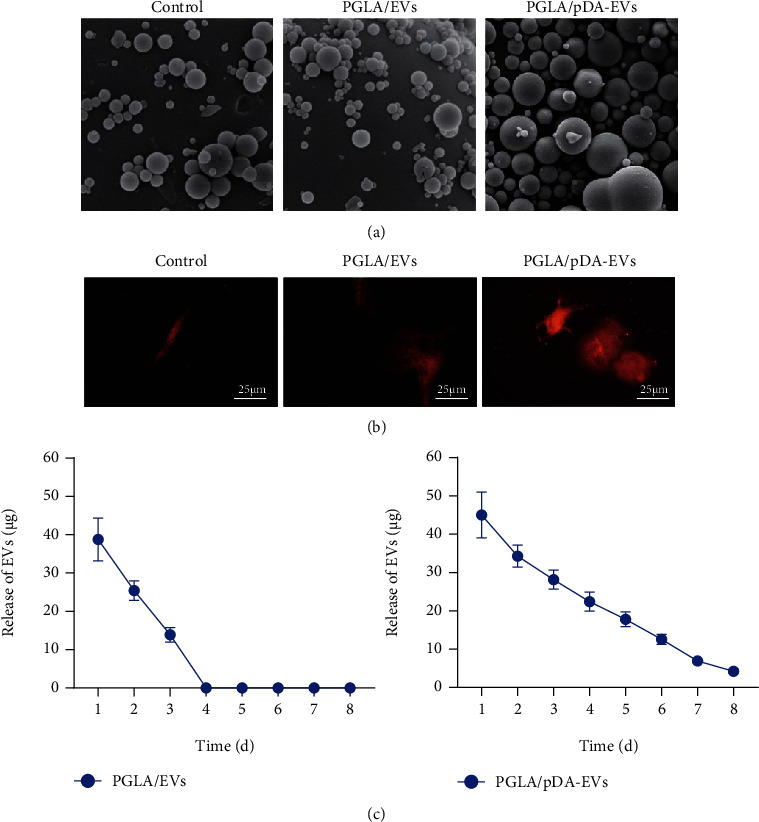
Construction and characterization of the PLGA/pDA-EV scaffold. (a) FESEM microscopic views of PLGA, PLGA/EVs, or PLGA/pDA-EVs. (b) Distribution of PKH26-labeled EVs on PLGA (middle) and PLGA/pDA scaffolds (right) observed under a laser confocal scanning microscope (scale bar = 25 *μ*m). PKH26 stained scaffold was used as a control (left). (c) Release of EVs from the PLGA/EVs or PLGA/pDA-EVs, *N* = 3.

In order to explore the biological effects of EVs, we used PLGA/pDA and hADSCs-EVs to construct a new type of cell-free TEB that mimics the paracrine function of cells. FESEM analysis confirmed that the surface morphology of different coating materials was significantly different. The EV particles distributed on the surface of the PLGA/pDA-EV scaffold were different from the polydopamine particles, being cup-shaped and refracted (Figure 5(a)). We discovered that EVs labeled by PKH26 (red dots) were evenly distributed on the scaffold surface, while when PKH26 staining was used as a control, there were only scattered irregular plaques on the scaffold. Compared with the PLGA scaffold, more red dots were seen on the PLGA/pDA scaffold (Figure 5(b)). The release kinetic analysis results showed that almost all EVs were released from the PLGA scaffold within 4 days. In contrast, during the 8-day monitoring period, the release of EVs from the PLGA/pDA scaffold was slow (Figure 5(c)). These findings indicate that the PLGA/pDA scaffold can load more EVs, which were released more slowly, and the PLGA/pDA-EV scaffold is suitable for in vivo experiments of stable release of EVs.

### 3.6. PLGA/pDA-EVs Promote the Repair of Alveolar Bone Defects in Rats by Delivering CGRP

**Figure 6:**
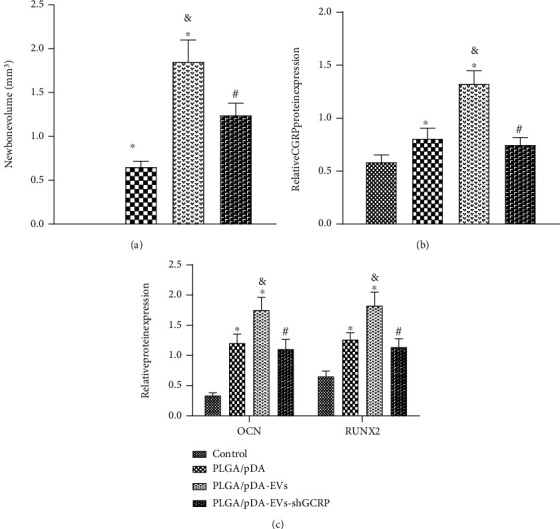
PLGA/pDA-EVs enhance the repair of alveolar bone defects in rats by delivering CGRP. Rats were treated with PLGA/pDA, PLGA/pDA-EVs, or PLGA/pDA-EVs-shGCRP. (a) The repair of alveolar bone defects of rats analyzed by micro-CT. (b) Western blot analysis of CGRP protein in the alveolar bone tissues of rats. (c) Western blot analysis of RUNX2, ALP and OCN proteins in alveolar bone tissues of rats. *n* = 10 for rats upon each treatment. ^∗^*p* < 0.05, compared with control rats. #*p* < 0.05, compared with PLGA/pDA-EV-treated rats. &*p* < 0.05, compared with PLGA/pDA-treated rats, *N* = 3.

Finally, we sought to determine whether PLGA/pDA-EVs regulate alveolar bone defects by delivering CGRP. The micro-CT results showed a small number of high-density spots in the alveolar bone of rats treated with EVs. Several high-density spots and small peninsula-shaped bone nodules were seen on the edge of the bone defect in the alveolar bone of PLGA/pDA-EVs-treated rats, while the bone defect repair was not obvious in the presence of PLGA/pDA-EVs-shGCRP (Figure 6(a), Supplementary Figure 3A), suggesting the superior promoting effect of PLGA/pDA-EVs on new bone formation.

An enhancement in the expression of CGRP was seen following treatment with PLGA/pDA-EVs while a contrary result was observed in response to treatment with PLGA/pDA-EVs-shGCRP (Figure 6(b), Supplementary Figure 3B).

Further HE staining results showed that the alveolar bone defects of the PLGA/pDA- and PLGA/pDA-EV-shGCRP-treated rats were mainly filled with fibrotic connective tissues. In contrast, in the presence of PLGA/pDA-EVs, the newly formed bone tissues could be observed at the edge and center of the alveolar bone defect (Supplementary Figure 3C). Masson's trichrome staining results displayed more mature collagen formed in the alveolar bone of PLGA/pDA-EV-treated rats, while PLGA/pDA-EV-shGCRP treatment led to contrary result (Supplementary Figure 3D).

Western blot analysis results indicated an upward inclination in the expression of ALP, RUNX2, and OCN following treatment with PLGA/pDA or PLGA/pDA-EVs but an opposite result was induced upon treatment with PLGA/pDA-EVs-shGCRP (Figure 6(c)). Thus, PLGA/pDA-EVs can facilitate the repair of alveolar bone defects in rats by delivering CGRP.

### 3.7. Discussion

The potential application of hADSC-EVs in the bone tissue regeneration has been largely reported [21, 22]. Here, we pinpointed the promoting effect of PLGA/pDA-loaded hADSC-EVs on the osteogenic differentiation ability of hPDLSCs and the ensuing repair of alveolar bone defects by delivering CGRP.

We initially found that CGRP was enriched in the hADSC-EVs. EVs contain many cargos that are important in cell-cell communication, including DNA, proteins/peptides, mRNAs, miRNAs, lipids, and organelles [23, 24]. CGRP has been confirmed to be a differentially expressed mRNA mainly involved in EVs [25]. In addition, exosomes have been found to be localized within CGRP^+^ sensory neurons [26]. Further analysis exhibited that hADSC-EVs promote the osteogenic differentiation behavior of hPDLSCs in vitro. hPDLSCs are a kind of mesenchymal stem cells with multiple differentiation capabilities isolated from periodontal ligament tissues and have broad application prospects in tissue regeneration [27, 28]. Consistently, the exosomes isolated from human exfoliated deciduous teeth can promote the osteogenic differentiation of PDLSCs with deep ARS staining, high ALP activity, and upregulated RUNX2, OPN, and OCN [29]. In addition, previous results have indicated that BMSC-derived EVs can elevate the expression of osteogenic genes and stimulate osteoblastic differentiation [30]. Meanwhile, the exosomes secreted by osteocytes contributed to hPDLSC osteogenic differentiation by regulating the BMP2/Runx2 axis [31]. We also demonstrated that the promoting effect of hADSC-EVs on the osteogenic differentiation property of hPDLSCs in vitro was related to the transfer of CGRP into hPDLSCs. In agreement with our results, a previous study has revealed that the overexpression of CGRP significantly promotes BMSC proliferation, upregulates the expression of osteogenesis-related indexes ALP, BSP, and RUNX2, and induces mineralization nodules [20]. CGRP, released by nerve fibers, can inhibit the apoptosis of human osteoblasts, thus favoring local bone regeneration [32]. Additionally, CGRP is able to induce CALCRL- and RAMP1-dependent activation of CREB1 and SP7, consequently increasing the osteogenic differentiation of the periosteum-derived stem cells [33]. Moreover, ADSCs transduced with adenoviral vectors carrying CGRP exhibit dramatically enhanced efficiency in the new bone formation in vivo during rat radial bone defects [9]. In addition, the osteogenic differentiation of hPDLSCs has been highlighted to be critical for alveolar bone regeneration [34]. The aforesaid data highlighted the use of CGRP delivered by hADSC-EVs in bone defect therapies.

Large bone defects represent predominate health concern worldwide, and TEB involving the utilization of hMSCs has recently pinpointed as a key modality for the regeneration of damaged bone tissues [35]. TEB based on MSCs at 6 months postgrafting in the atrophic maxilla has shown a statistically significant increase in new bone formation, accompanied by few residual graft particles and connective tissues [36]. In the current study, we employed PLGA/pDA and hADSC-EVs to construct a new type of TEB based on MSCs and implanted it into the alveolar bone defects in rats to test the effect of PLGA/pDA-EVs on the repair of alveolar bone defects. The obtained results demonstrated that PLGA/pDA-EVs slowed the release of EVs from the PLGA/pDA-EV scaffold and induced the repair of alveolar bone defects in rats by delivering CGRP. A recent study revealed that the PLGA/nanohydroxyapatite/chitosan (nHA/CS)/rhBMP-2 scaffold effectively controls the early burst effect of rhBMP-2 and exerts osteogenic effects, thus accelerating the repair of the experimental bone defect area of the rabbit mandible [37]. The mussel-inspired gold nanoparticle and PLGA/L-lysine-g-graphene oxide composite scaffolds can significantly improve osteogenic differentiation in vitro and induce the formation of the new bone and collagen deposition in the radial defect site [38]. A cell-free system comprised of hADSC-derived exosomes and PLGA/pDA scaffold can significantly enhance bone regeneration, which is partially through its osteoinductive effects found in the newly formed bone tissues [27]. In addition, a constructed delivery system composed of HyStem-HP hydrogel and EVs can substantially promote bone formation in rats with calvarial defects [30]. Importantly, the PLGA nanoparticle is responsible for the sustainable release of CGRP, and the polymer-based CGRP delivery system may drive the therapeutic effects of the CGRP on vascular and inflammatory disorders [11]. Based on these results, the combination of PLGA/pDA-EV scaffold and CGRP shows considerable potential to accelerate the repair of alveolar bone defects.

## 4. Conclusions

**Figure 7:**
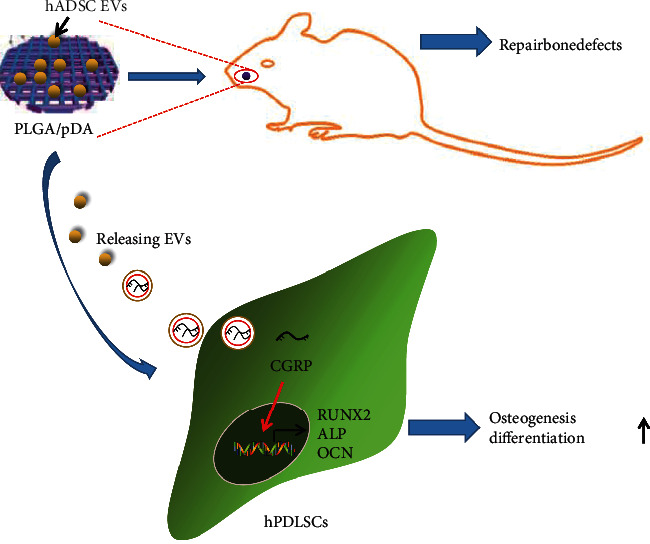
Schematic diagram of the mechanism by which PLGA/pDA-loaded hADSC-EVs affect the osteogenic differentiation of hPDLSCs. PLGA/pDA-loaded hADSC-EVs may promote the osteogenic differentiation of hPDLSCs by delivering CGRP, thereby promoting the repair of alveolar bone defects.

In conclusion, we pinpointed that PLGA/pDA loaded hADSC-EVs promoted the osteogenic differentiation ability of hPDLSCs in vitro and the ensuing repair of alveolar bone defects in vivo via transfer of CGRP (Figure 7). These findings may provide new therapeutic modalities for bone tissue regeneration. However, due to the lack of available literature regarding the implication of CGRP in hPDLSCs, further investigation is a prerequisite to validate the obtained results. In addition, further studies are also required based on the clinical samples to reproduce and provide statistical support for our conclusion.

## Data Availability

The datasets generated/analyzed during the current study are available.

## Supplementary Materials

Supplementary Materials Supplementary Figure 1: quality control analysis of the hPDLSC osteogenic differentiation-related gene expression dataset GSE53929. (a) A volcano map of the differential analysis results on the GSE53929 dataset. The red dots indicate the highly expressed genes in the treated group, the blue dots indicate the poorly expressed genes, the abscissa indicates log2 (fold change), and the ordinate indicates -log10 (*p* value). (b) UMAP plot of the sample. (c) A box plot of screened genes from the GSE53929 dataset in the control and treated groups. (d) Expression density plot of genes from the GSE53929 dataset in the control and treated groups. (e) *p* value histogram. (f) *t*-statistic quantile plot. Supplementary Figure 2: western blot analysis of hPDLSC surface markers CD73, CD45, and CD90 in the hPDLSCs. Supplementary Figure 3: effect of PLGA/pDA-EVs on the repair of alveolar bone defects in rats. (a) Micro-CT observation of the repair of the alveolar bone defect of rats after 6 weeks. (b) Immunofluorescence staining analysis of CGRP protein in the alveolar bone semithin section sample (scale bar = 50 *μ*m). (c) HE staining analysis of rat alveolar bone formation (scale bar = 50 *μ*m). (d) Masson's trichrome staining of collagen content in rat alveolar bone matrix (scale bar = 50 *μ*m). Supplementary Table 1: primer sequences for reverse transcription quantitative polymerase chain reaction. Supplementary Table 2: overlapped genes of the differentially highly expressed genes from the GSE53929 dataset and the top 100 genes related to hPDLSC osteogenic differentiation from the GeneCards database.
