# Supplementary material for: PLGA Containing Human Adipose-Derived Stem Cell-Derived Extracellular Vesicles Accelerates the Repair of Alveolar Bone Defects via Transfer of CGRP
Source: Oxid Med Cell Longev. 2022 Jun 11;2022:4815284. doi: 10.1155/2022/4815284 (PMC9206573; doi:10.1155/2022/4815284)
Supplement: Supplementary Materials — Supplementary Figure 1: quality control analysis of the hPDLSC osteogenic differentiation-related gene expression dataset GSE53929. (a) A volcano map of the differential analysis results on the GSE53929 dataset. The red dots indicate the highly expressed genes in the treated group, the blue dots indicate the poorly expressed genes, the abscissa indicates log2 (fold change), and the ordinate indicates -log10 (p value). (b) UMAP plot of the sample. (c) A box plot of screened genes from the GSE53929 dataset in the control and treated groups. (d) Expression density plot of genes from the GSE53929 dataset in the control and treated groups. (e) p value histogram. (f) t-statistic quantile plot. Supplementary Figure 2: western blot analysis of hPDLSC surface markers CD73, CD45, and CD90 in the hPDLSCs. Supplementary Figure 3: effect of PLGA/pDA-EVs on the repair of alveolar bone defects in rats. (a) Micro-CT observation of the repair of the alveolar bone defect of rats after 6 weeks. (b) Immunofluorescence staining analysis of CGRP protein in the alveolar bone semithin section sample (scale bar = 50 μm). (c) HE staining analysis of rat alveolar bone formation (scale bar = 50 μm). (d) Masson's trichrome staining of collagen content in rat alveolar bone matrix (scale bar = 50 μm). Supplementary Table 1: primer sequences for reverse transcription quantitative polymerase chain reaction. Supplementary Table 2: overlapped genes of the differentially highly expressed genes from the GSE53929 dataset and the top 100 genes related to hPDLSC osteogenic differentiation from the GeneCards database. [file 4815284.f1.docx]

**Supplementary Table 1** Primer sequences for reverse transcription quantitative polymerase chain reaction

| Gene | Primer sequence |
| --- | --- |
| ALP (human) | Forward 5’-GTGAACCGCAACTGGTACTC-3’ |
|  | Reverse 5’-GAGCTGCGTAGCGATGTCC-3’ |
| OCN (human) | Forward 5’-CACTCCTCGCCCTATTGGC-3’ |
|  | Reverse 5’-CCCTCCTGCTTGGACACAAAG-3’ |
| RUNX2 (human) | Forward 5’-TGGTTACTGTCATGGCGGGTA-3’ |
|  | Reverse 5’-TCTCAGATCGTTGAACCTTGCTA-3’ |
| GAPDH (human) | Forward 5’-AGCCACATCGCTCAGACAC-3’ |
|  | Reverse 5’-GCCCAATACGACCAAATCC-3’ |

**Supplementary Table 2** Overlapped genes of the differentially highly expressed genes from the GSE53929 dataset and the top 100 genes related to hPDLSC osteogenic differentiation from the GeneCards database

| Number | Gene |
| --- | --- |
| 1 | FGFR2 |
| 2 | MET |
| 3 | SMAD6 |
| 4 | TNFRSF11A |
| 5 | MIR21 |
| 6 | DLX5 |
| 7 | NOTCH3 |
| 8 | KRT1 |
| 9 | TGFB3 |
| 10 | SMAD4 |
| 11 | BMP4 |
| 12 | NOTCH1 |
| 13 | MEN1 |
| 14 | ALPP |
| 15 | SPP1 |
| 16 | GNAS |
| 17 | PTH1R |
| 18 | DMP1 |
| 19 | EGF |
| 20 | CDK4 |
| 21 | FGF23 |
| 22 | TNFSF11 |
| 23 | SP7 |
| 24 | PPARG |
| 25 | ESR1 |
| 26 | CYP27B1 |
| 27 | IBSP |
| 28 | NF1 |
| 29 | SOST |
| 30 | CALCA |
| 31 | MAPK1 |
| 32 | MYC |

**
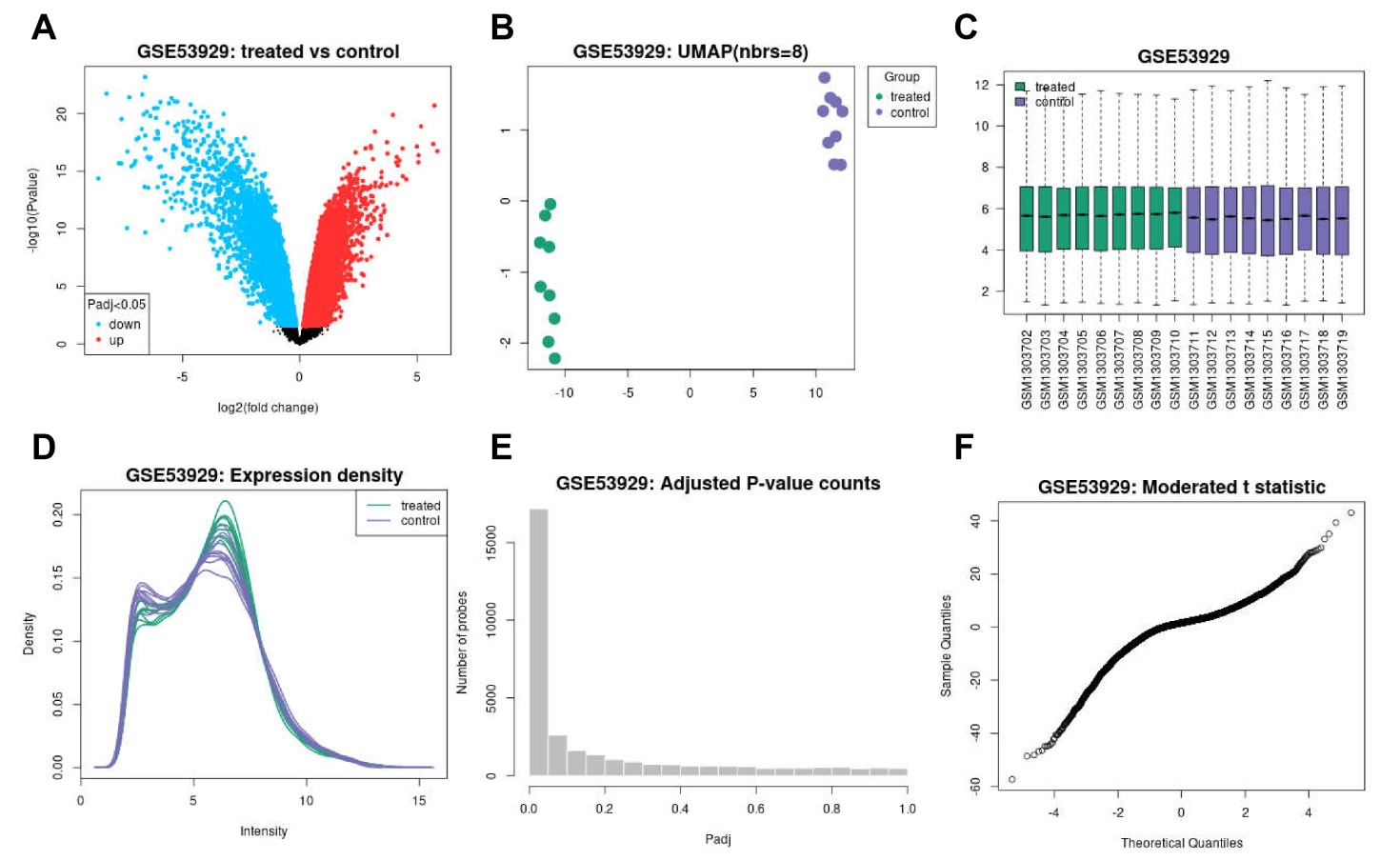
**

**SUPPLEMENTARY FIGURE 1:** Quality control analysis of the hPDLSC osteogenic differentiation-related gene expression dataset GSE53929. A, A volcano map of the differential analysis results on the GSE53929 dataset. The red dots indicate the highly expressed genes in the treated group, the blue dots indicate the poorly expressed genes, the abscissa indicates log2 (fold change), and the ordinate indicates -log10 (*p* value). B, UMAP plot of the sample. C, A box plot of screened genes from the GSE53929 dataset in the control and treated groups. D, Expression density plot of genes from the GSE53929 dataset in the control and treated groups. E, *p* value histogram. F, t-statistic quantile plot.

**
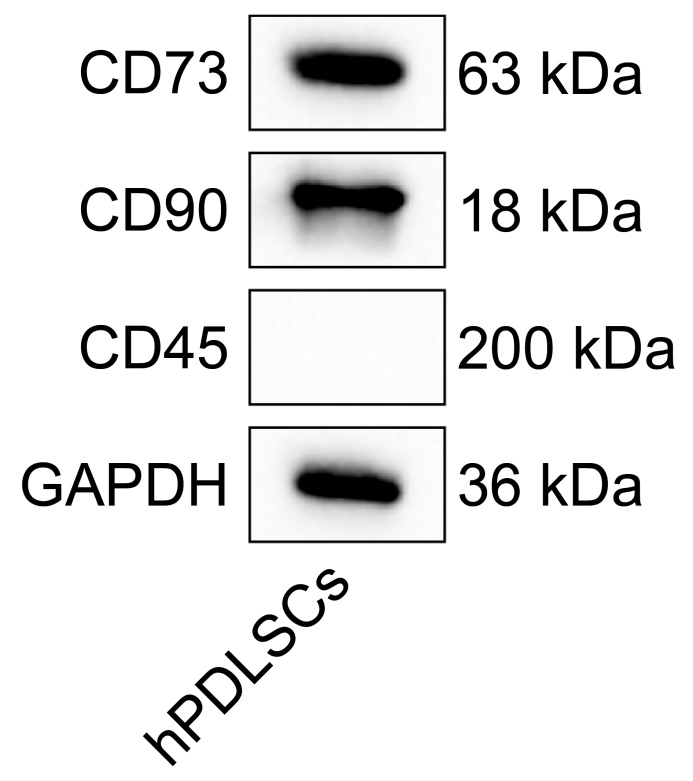
**

**SUPPLEMENTARY FIGURE 2:** Western blot analysis of hPDLSC surface markers CD73, CD45 and CD90 in the hPDLSCs

**
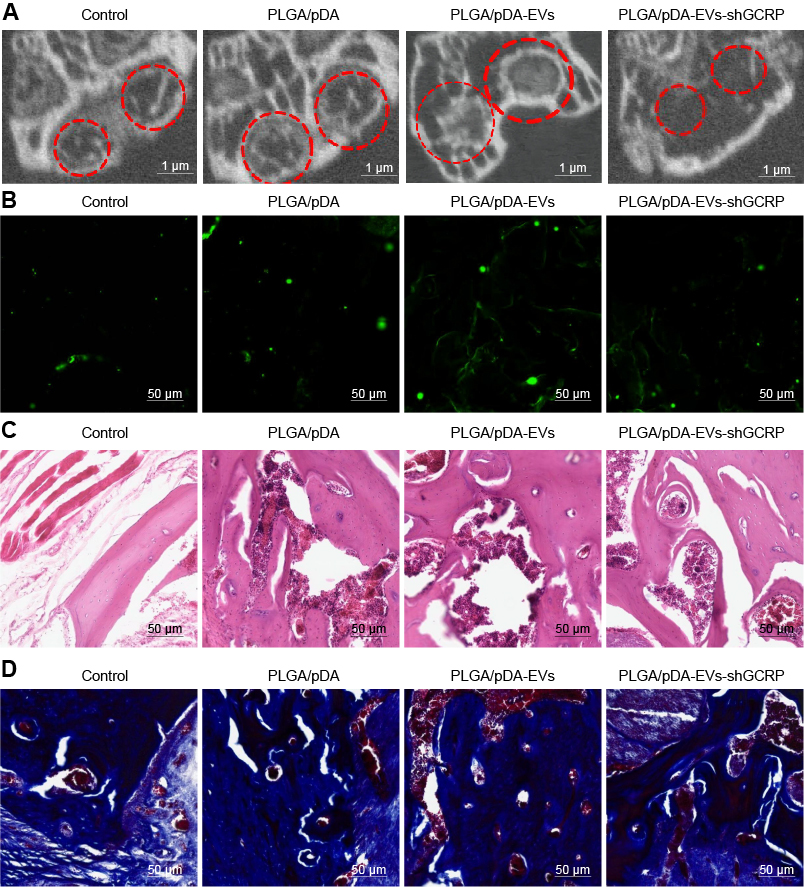
**

**SUPPLEMENTARY FIGURE 3:** Effect of PLGA/pDA-EVs on the repair of alveolar bone defects in rats. A, Micro-CT observation of the repair of the alveolar bone defect of rats after 6 weeks. B, Immunofluorescence staining analysis of CGRP protein in the alveolar bone semi-thin section sample (scale bar = 50 μm). C, HE staining analysis of rat alveolar bone formation (scale bar = 50 μm). D, Masson’s trichrome staining of collagen content in rat alveolar bone matrix (scale bar = 50 μm).
